# The domestication of the larynx: The neural crest connection

**DOI:** 10.1002/jez.b.23251

**Published:** 2024-04-09

**Authors:** Raffaela Lesch, W. Tecumseh Fitch

**Affiliations:** 1 Department of Biology, University of Arkansas at Little Rock, Little Rock, Arkansas, USA; 2 Department of Behavioral and Cognitive Biology, University of Vienna, Vienna, Austria

**Keywords:** bioacoustics, development, sound production, tameness, voice

## Abstract

Wolves howl and dogs bark, both are able to produce variants of either vocalization, but we see a distinct difference in usage between wild and domesticate. Other domesticates also show distinct changes to their vocal output: domestic cats retain meows, a distinctly subadult trait in wildcats. Such differences in acoustic output are well-known, but the causal mechanisms remain little-studied. Potential links between domestication and vocal output are intriguing for multiple reasons, and offer a unique opportunity to explore a prominent hypothesis in domestication research: the neural crest/domestication syndrome hypothesis. This hypothesis suggests that in the early stages of domestication, selection for tame individuals decreased neural crest cell (NCCs) proliferation and migration, which led to a downregulation of the sympathetic arousal system, and hence reduced fear and reactive aggression. NCCs are a transitory stem cell population crucial during embryonic development that tie to diverse tissue types and organ systems. One of these neural-crest derived systems is the larynx, the main vocal source in mammals. We argue that this connection between NCCs and the larynx provides a powerful test of the predictions of the neural crest/domestication syndrome hypothesis, discriminating its predictions from those of other current hypotheses concerning domestication.

## 1 Introduction: Voice Production and Domestication

Asked to name sounds specific to a domestic animal, many people would immediately think of cat meows and dog barks. Cat meows are mostly used in cat-human interactions by adult domestic cats, but are not typically observed in wild adult cat-cat interactions (Bradshaw & Cameron-Beaumont, 2000; Nicastro, 2004). Similarly, dogs produce barks in a wide variety of different contexts and scenarios, while in wolves, the bark is much less ubiquitous, has a different acoustic structure, and a very specific usage in threatening contexts (Faragó et al., 2013). However, with cat meows and dog barks, we see only the end product of millennia of domestication, and can only speculate about the potential causes of these peculiarities.

**Figure 1:**
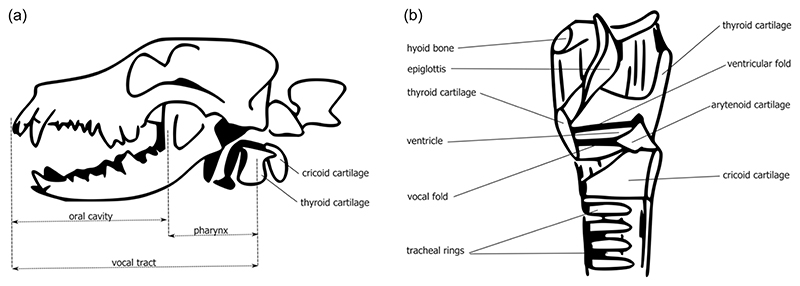
(a) Position of the mammalian larynx relative to the skull in a dog. (b) Midsagittal cross-section with individually labeled components of mammalian laryngeal structure. This portion of the schematic (based on a human larynx) is adapted from Titze (1994).

While barks are strongly associated with dogs, basenjis (an ancient dog breed from Africa) are known for not being able to bark but instead “yodel” (Ashdown & Lea, 1979). We know little about the mechanistic basis of this phenomenon, but research on basenji laryngeal structure found that the ventricles are shallow in comparison to other dog breeds (for an overview of vocal anatomy, see Figure 1). This change in ventricle depth has been argued to underlie the basenji’s inability to produce explosive bark vocalizations (Ashdown & Lea, 1979). New Guinea singing dogs are also known for their strongly modulated howl, and a high-frequency pulsed trill which has been hypothesized to be produced by rapid vibration of a rudimentary uvula not found in other canids (Koler-Matznick et al., 2003). For these examples, it is unclear whether these vocal traits were specifically selected for by humans, if they are a by-product of domestication, or result from some other unknown type of selection pressure.

Research directly evaluating the impact of domestication on voice production is sparse, especially in mammals. However, several studies have investigated the vocal output of domesticated foxes, selected specifically and solely for tameness (see below), in comparison to their unselected-type cousins, and clearly tie changes in vocal output to the domestication process itself. For example, Gogoleva et al. (2008) compared domesticated foxes to unselected and aggressive foxes, finding that domesticated foxes produce a series of explosive vocalizations when greeting humans (Gogoleva et al., 2011). A crossbreeding experiment further suggested that these human-directed vocalizations may represent a discrete phenotypic trait of tame foxes (Gogoleva et al., 2009). We are aware of no studies of laryngeal morphology in these foxes.

To our knowledge, other research and comparative approaches to date cannot rule out that observed changes to the voice and vocalizations in domesticated species were a result of direct breeding rather than side-effects of domestication. While changes to the vocal production system due to recent line/pedigree breeding are not directly informative about early domestication processes, they nonetheless allow potentially interesting mechanistic insights. In the remainder of this manuscript we mainly focus on the measurable impact of domestication on the mammalian larynx and vocal tract.

### 1.1 Neural crest and the domestication syndrome

Starting with a hypothesis that tameness was a crucial factor in the early process of domestication, Dmitri Belyaev et al. initiated a daring experiment in the 1950s (Francis, 2015; Trut et al., 2009): to attempt to domesticate a novel species within a human lifetime. Belyaev’s team began selecting fur farm foxes based solely on their objectively measured tameness toward humans, as evaluated in young foxes. Remarkably, within six generations they observed additional, unselected changes in both behavior (e.g., tail wagging) and morphology (e.g., white spots, floppy ears, and curly tails). The experiment continues today, and after generation 42, unusual traits commonly seen in domesticated dogs are now widespread among the tame fox population (Kukekova et al., 2014). This experiment has since been successfully repeated with wild-caught rats (*Rattus norvegicus*), again selecting solely for tameness, and again resulting in unselected changes to the behavior, physiology, and neurochemistry (Albert et al., 2008). These studies strongly support the idea that selection for tameness alone is enough to initiate the domestication process, and leads to other correlated changes as a byproduct (Trut et al., 2009).

This finding is intriguing, because diverse domesticated animal species show unusual overlapping changes in behavior and morphology (Hemmer, 1990; Kukekova et al., 2014). Domesticated animals typically show great variability in appearance compared to wild populations, but individuals or breeds often show traits rare or absent in wild populations, including white spots, floppy ears, smaller cranial capacities, shorter muzzles, smaller teeth, and curled tails (Sánchez-Villagra et al., 2016; Wilkins et al., 2014). This rather bizarre collection of unusual traits was first catalogued by Darwin, and more recently has been dubbed the “domestication syndrome” by Wilkins et al. and Hare et al. (Darwin, 1868; Hare et al., 2012; Wilkins et al., 2014, 2021). Although these changes are well documented in particular breeds of a diverse set of species, a unified explanation for their common occurrence was lacking.

The neural crest/domestication syndrome (NCDS) hypothesis by Wilkins et al. (2014, 2021) offers such a unifying explanation, aiming to explain all of these diverse traits under one over-arching developmental/mechanistic explanation, hypothesizing that a mild decrease in neural crest cell (NCCs) proliferation and migration could generate nearly all of traits included in the domestication syndrome (Pendleton et al., 2018; Torres-Pérez et al., 2023; Wilkins et al., 2014, 2021). NCCs are a transitory stem cell population that emerges from the region of the neural tube during early stages of embryogenesis (Eames et al., 2020) and then disperse throughout the body. NCCs are specific to vertebrates, where they represent a crucial innovation in early vertebrate evolution (Gans & Northcutt, 1983), acting as precursors to an impressive variety of cell types and tissues throughout the body, and are further implicated in the development of many others. Intriguingly, many of the other tissues connected to the NCCs (including pigmentation cells, facial skeleton, teeth, and ear cartilages) are affected in the domestication syndrome across a wide range of domesticated species. Crucially, the entire sympathetic nervous system, along with key components of the adrenal glands, are derived from NCCs, and a downregulation of this system could increase tameness (reduced fear and reactive aggression toward humans; Gilbert & Barresi, 2016, see pp 465–466). The NCDS hypothesis suggests that during the early stages of domestication, largely unconscious selection for tame individuals was achieved mechanistically by downregulating NCCs proliferation and migration, which in turn led to diminution and delayed maturation of the sympathetic arousal system, and thus to general reduction and delayed onset of fearfulness and reactive aggression. The other traits of the domestication syndrome are hypothesized to result as unselected by-products of this downregulation of the neural crest under selection for tameness.

### 1.2 NCCs and the development of the larynx

**Figure 2:**
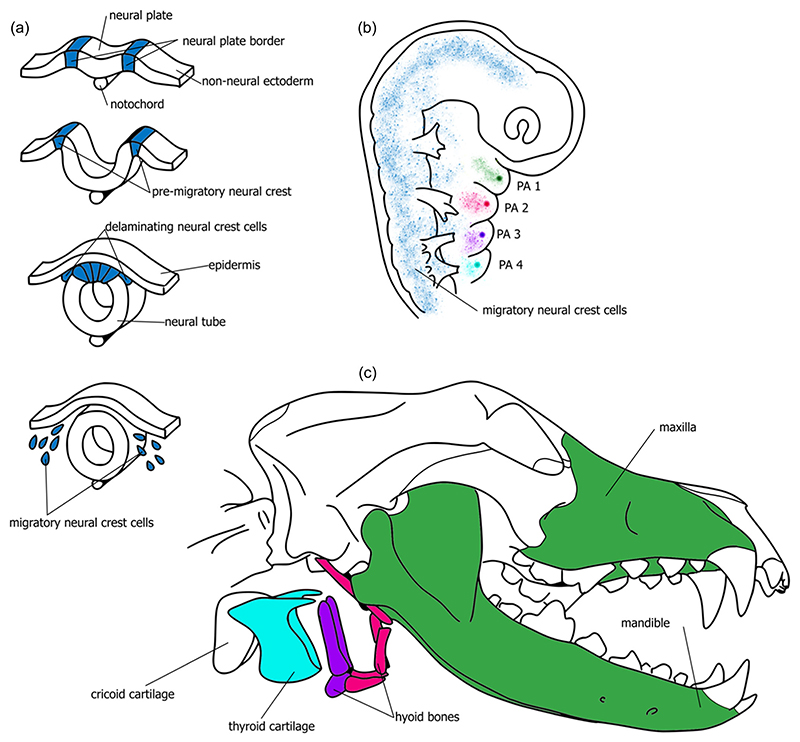
Illustration of the connection between the neural crest cell (NCCs) proliferation and migration and larynx development. (a) Schematic of the neurulation process and the origin and movement/migration of the NCCs. This portion of the schematic is adapted from Simões-Costa and Bronner (2015). (b) Schematic of the NCCs migration into the pharyngeal arches during embryonic development; color codes indicate pharyngeal arch number. This portion of the figure is adapted from Gilbert and Barresi (2016). (c) Schematic of the larynx and facial skeleton in relation to an adult dog skull. The jaws, hyoid, and larynx are color coded for their pharyngeal arch of origin. Pharyngeal arch one is indicated as green, PA two is indicated as magenta, PA three as purple, and PA four as turquoise. Neural crest contributes strongly to the thyroid cartilage (turquoise), a mammal-specific laryngeal cartilage. The cricoid and arytenoid cartilages that make up the rest of the mammalian larynx skeleton, and the entire larynx in other tetrapods, are not neural crest derived (Tabler et al., 2017). PA, pharyngeal arch.

NCCs are not just relevant for traits of the “domestication syndrome” described above, but are also crucial in the formation of key mammalian sound producing structures (Tabler et al., 2017): the larynx and vocal tract. The vocal tract consists of the supra-laryngeal oral, pharyngeal, and nasal cavities, ensconced within the facial skeleton which is nearly entirely neural-crest derived. The larynx is composed of the thyroid, cricoid, paired arytenoids, and epiglottic cartilages, along with the hyoid bones, and muscle and tissue derived from the pharyngeal (also called branchial) arches (Harrison, 1995b; Schneider, 1964; Figure 1). The developmental origins of the upper and lower jaws are found in the first pharyngeal arch, while the hyoid apparatus derives from the second and third arches. Traditionally, the larynx has been thought to originate from the second & third arch and the fourth and sixth (or seventh, depending on numbering convention) pharyngeal arches (Edgeworth, 1916; Gegenbauer, 1892; Harrison, 1995a; Wilder, 1892). Branchial arches four and six are crucial for the development of the thyroid cartilage, vocal folds, and perhaps other laryngeal cartilages (Danowitz et al., 2016; Figure 2). More specifically, the thyroid cartilage is said to develop from arch four, and the cricoid and arytenoid cartilage from arch six (Som & Curtin, 2014). All of these vocal structures were previously thought to have a major neural-crest-derived component. However, more recent cell-lineage tracing studies strongly suggest that while portions of the vocal folds and almost the entire thyroid cartilage are formed from NCCs, the cricoid and arytenoid are not (Laitman et al., 2017; Lungova & Thibeault, 2020; Tabler et al., 2017). Furthermore, an abundance of NCCs results in a degenerated larynx (Tabler et al., 2017). In summary, NCCs are crucial for the “correct” formation of the mammalian larynx and vocal tract.

This direct relationship between NCCs and laryngeal and vocal tract development combines with the putative connection between NCCs and domestication to raise fascinating questions at the intersection of bioacoustics and domestication research. First, did the domestication process change the larynx (and thus the voices) of domesticated animals? If so, could this be a by-product of initial selection for tameness, as posited by the NCDS?

## 2 Laryngeal Morphology As A Test Case For The Ncds

The central role of the neural crest in the formation of the larynx and vocal tract leads to a clear and novel prediction if the NCDS hypothesis. If the NCDS hypothesis is correct we should observe changes in vocal anatomy, and particularly in the neural-crest-derived jaws, hyoid apparatus, and thyroid cartilage, when comparing domesticated species with their wild-type ancestors. Although a modification of the jaws and teeth is already well-documented in some domesticates (Sánchez-Villagra et al., 2016), to our knowledge the anatomy of the hyoid and larynx has never been systematically compared for domesticates. The straightforward prediction would be that the hyoid apparatus and some components of the larynx (and in particular the thyroid cartilage and the vocal ligament within the vocal folds) should be significantly smaller in domesticates, however Tabler et al.’s finding that overabundance of NCCs led to *reduction* in laryngeal structures suggests that a more general change in shape and size (whether larger or smaller) should be expected.

How would these morphological changes affect the vocal output? At least some of the vocal changes discussed earlier in dogs and cats may result from these predicted changes in vocal morphology. Of course, other changes in vocal behavior may result from central nervous system changes, unrelated to neural crest and thus variable across domesticated species, and/or to changes in emotional arousal, due to changes in the sympathetic nervous system that may be more consistent across domesticates. We suggest that the selection for tameness can have a twofold impact on animal vocalizations: first, the connection between the neural crest and the larynx can impact sound structures connected to the physics of voice production (e.g., shorter vocal folds lead to a higher pitched voice), and second, sound production is also tied to animal behavior therefore domesticated animals might also produce altered vocalizations due to changes in behavior or cognition. This second aspect is supported by the avian data discussed below, because the avian syrinx is not derived from neural crest. Such neurobehavioral changes are also much more difficult to evaluate empirically, while hypotheses positing morphological changes can be evaluated with relative ease by direct anatomical comparisons. Thus, we hypothesize that vocal changes may constitute a novel component of the domestication syndrome, predicted by the NCDS, with changes in vocal morphology being the most readily testable prediction.

## 3 Avian Vocalization and Domestication

Several studies of the effects of domestication on avian sound production provide valuable insights to this problem, due to major differences in vocal production between birds and mammals. Birds produce sound through their syrinx, a sound production system that evolved independently in birds. The syrinx is solely responsible for sound production (unlike the mammalian larynx, which also plays a primary role in airway protection), and does not appear to be neural crest derived (Clarke et al., 2016; Kingsley et al., 2018). However, song variation has been related in several studies to beak morphology (Demery et al., 2021; Sakata et al., 2020), and the entire jaw is neural-crest derived in all tetrapods. Nonetheless, we view most of the findings from birds as providing examples of how domestication can modify neural and behavioral aspects of vocalization independent of major changes in the vocal apparatus per se.

Perhaps the best studied nonmammalian example directly demonstrating the impact of domestication on voice production involves the Bengalese finch. Bengalese finches are a domesticated songbird breed originating from the white-rumped munia, a passerine bird endemic to southeast Asia. Bengalese finches have been bred in captivity in Japan for over 240 years, during which they have been domesticated to their current all-white form that thrives in captivity (Okanoya, 2004). In the process of domestication these finches were selected not for their song, but for fecundity in captivity. Nonetheless, their song changed from a very simple and repetitive song found in white-rumped munias to the more complex and variable song form observed in the Bengalese finches (Okanoya, 2017).

In canaries, a passerine bird originating from the Canary islands, one study compared three different lineages: wild canaries, one line bred for their morphology, and one for their song (Güttinger, 1985). These selected lines offered the opportunity to study both the general impact of domestication and the specific impact that artificial selective breeding for a trait has on song (however, note that the sound producing organ in birds is the syrinx, not the larynx). The study found that the duration of song “tours” and proportion of single utterances in German Roller canaries changed during the course of domestication. The author suggested that selective breeding favors the augmentation of traits that are already present as byproducts of domestication (Güttinger, 1985). In canaries, domesticated lineages that have only been bred for morphology already exhibit the same trends observed in lineages bred for song. Güttinger (1985) thus suggested that traits can only be selected for during artificial selection if they are already present to some extent in the original domesticated group.

What these examples demonstrate is that acoustic changes to vocal output can occur with domestication, independently of any known differences in the vocal production apparatus itself. Thus, these changes presumably stem from neurobehavioral differences, rather than vocal morphological differences, in domesticates relative to wild type forebears. These findings thus support our proposal to focus initially on easily evaluated vocal morphological changes as a first step to testing the NCDS.

### 3.1 Testing multiple hypotheses about domestication using vocal morphology

The NCDS hypothesis is not uncontested, and multiple other possibilities have been suggested to account for changes seen under domestication (Gleeson & Wilson, 2023; Lord, Larson, Coppinger, et al., 2020; Lord et al., 2020a). We now will briefly review these other possibilities, considering how research into the connection between the larynx and neural crest could be highly relevant in testing between the NCDS hypothesis and the other currently plausible hypotheses.

I) Pre-domestication? Lord et al. have criticized the Russian domesticated fox experiments, pointing out that the animals initiating this experiment had already undergone many years of captive breeding for their fur, and thus might already have been “pre-domesticated” (Lord, Larson, Coppinger, et al., 2020; Lord et al., 2020a). While this is plausible, this point of history was already familiar to the Russian team (Statham et al., 2011; Trut et al., 2020), who consistently examined effects of selection for tameness after this timepoint, relative to unselected control lines from the same founder population. Thus, the changes documented in the domesticated foxes are in contrast to control foxes derived from the same lineage. In any case, this observation concerning foxes would not negate the existence of the domestication syndrome, that is, common morphological and anatomical changes under domestication in other species (Zeder, 2020). Lord et al. (2020b) further argue that more rigorous approaches are required to test the predictions derived from the NCDS hypothesis; we agree, and one such approach testing novel predictions about vocal and laryngeal morphology is advanced here. We predict changes in vocal/laryngeal morphology in domesticated foxes relative to unselected lines, which controls for potential founder effects. The Lord et al. “preselection” hypothesis thus does not predict significant changes in laryngeal morphology.
II) Reproductive disruption: A recent paper by Gleeson and Wilson (2023) suggests that consistent disruptions to reproductive strategies provide an alternative explanation to the NCDS hypothesis for understanding the traits of the domestication syndrome. These authors suggest two somewhat independent main pathways to reproductive disruption as a response to the captive niche: disrupted sexual selection (mainly males) or an altered reproductive niche for females. Both these pathways describe an adaptation to a new niche, that is, either a human environment or any environment that favors selection for tame behaviors (e.g., islands without predators). While it is a valid point that such adaptation to a new environment may cause changes to reproduction, reproductive changes and selection pressures for tameness are not mutually exclusive, and as suggested by Belyaev, changes to reproductive strategies could represent a downstream effect of selection for tame behavior; either due to a human environment or any environment with active selection pressures toward “tame” or friendly behavior. However, the NCDS predicts changes in laryngeal morphology that are not predicted by the “reproductive disruption” hypothesis.
III) Thryoid disregulation: The thyroid hormone hypothesis provides another alternative hypothesis positing a direct connection between domestication and development. It was first proposed by Crockford and was named as such by Wilkins (Crockford, 2002; Wilkins, 2017). This hypothesis suggests that domestication might have shifted timing in general development which in turn would have the potential to affect the concentration of thyroid hormones during the developmental process. Thyroid hormones play a role in the development of the larynx and craniofacial structures in at least some species. For example, deficiencies of thyroid hormones affect ossification of the human skull (Leitch et al., 2020) and laryngeal development and differentiation in Xenopus frogs (Robertson & Kelley, 1996). While the thyroid hypothesis doesn’t provide an explanation for the entire collection of the traits comprising the domestication syndrome, it may help to explain certain observed changes in cranial structures (like snout reduction) that are linked to voice producing structures. However, despite their shared name, the thyroid gland and thyroid cartilage have independent embryological origins: modern lineage-tracing data show that the thyroid gland stems almost entirely from endoderm, with only its connective tissue sheathing stemming from neural crest (Nilsson & Fagman, 2017), overturning an old but well-entrenched notion that this gland was neural-crest derived (Nilsson & Williams, 2016).
  Hypothyroidism (underactive thyroid gland) has been connected to hoarseness of the voice in humans (Altman et al., 2003) and arrested development of the larynx in *Xenopus* (Robertson & Kelley, 1996). This suggests that changes in thyroid hormones could impact the laryngeal morphology, as predicted by the NCDS hypothesis. Yet on closer inspection the impact of the thyroid hormones is distinctly different from the proposed impact of the neural crest. In mammals, Ritter (1964) artificially induced complete hypothyroidism in rats and observed relatively subtle changes the submucosal portions of the vocal folds, yet found that both the vocal fold diameter and overall laryngeal structures were unaffected. Altman et al. (2003) further investigated the presence of thyroid hormone receptors in the larynx through antibody staining. They found consistent and strong evidence for receptors in the connective tissue of the lamina propria of the vocal folds with only light staining in the laryngeal cartilages. Both studies suggest that details of the vocal folds may be affected by an underactive thyroid gland.
  In frogs, Robertson and Kelley (1996) found that thyroid hormone blockers inhibited the development of the larynx in metamorphosing *Xenopus* larvae. Larvae with induced hypothyroidism exhibited “low density and minimal patterning of chondrocytes” and an “interference with myelination of the laryngeal axons.” They also demonstrated that an underactive thyroid gland led to both a larger larynx and body mass. While these results from frogs indicate subtle influences of thyroid hormones on laryngeal function, they do not predict changes in shape and size of the laryngeal cartilages in mammals, as predicted by the NCDS hypothesis.

In summary, to our knowledge, the NCDS hypothesis is the only current hypothesis seeking to explain the domestication syndrome that predicts morphological changes in laryngeal anatomy in domesticates. These predictions thus provide a strong test case for the NCDS.

### 3.2 A potential link to vocal changes in self-domestication?

The human self-domestication hypothesis suggests that certain human-specific traits are an outcome of evolutionary selection pressures similar to those present during animal domestication, namely reduced reactive aggression (Hare et al., 2012). Humans are not the only species suggested to have gone through the process of self-domestication, and both bonobos and elephants are argued to show traits consistent with the domestication syndrome (Hare et al., 2012; Raviv et al., 2023; Summers & Summers, 2023). Although the NCDS is focused solely on “true” domesticated species, its mechanistic basis has also been suggested to apply to humans and bonobos (Benítez-Burraco & Kempe, 2018; Theofanopoulou et al., 2017).

Consistent with this fusion of the two distinct hypotheses, comparative research on the bonobo and chimpanzee larynx found that bonobos have shorter vocal folds than chimpanzees (Grawunder et al., 2018), and that these shorter vocal fold lengths produce increased fundamental frequencies (i.e., higher voice pitch). A smaller overall larynx in bonobos would explain these documented shorter vocal fold lengths, consistent with the NCDS hypothesis if the supposition of bonobo self-domestication is accepted. It would thus be useful to provide more detailed comparisons of other aspects of laryngeal morphology, especially the size and shape of the hyoid apparatus and thyroid cartilages. Documented simplifications of laryngeal anatomy in humans (Nishimura et al., 2022) might also be seen as consistent with the NCDS and human self-domestication hypotheses.

## 4 Summary and Conclusions

Because of their (partial) derivation from the neural crest, the jaws, hyoid apparatus, and larynx provide us with the unique possibility of addressing all currently popular hypotheses for domestication syndrome with one empirical approach. Looking at a morphological system so clearly interwoven with the NCCs provides a powerful test of the predictions of the NCDS hypothesis, capable of discriminating it from other hypotheses. The NCDS hypothesis predicts fewer NCCs arriving at the site of the larynx, therefore leading to morphological changes in the larynx in domesticates compared to their wild counterparts. To our knowledge, none of the other hypotheses available make this prediction. If the hyoid and larynx are not affected by domestication, this would constitute evidence against the idea that domestication and the neural crest are linked, as posited by the NCDS hypothesis. Given the relative ease of obtaining specimens of the hyoid and larynx for a variety of domesticates and their wild-type cousins, this is a readily tested prediction, and we are beginning to acquire the required sample for dogs, cats, and pigs in our own laboratories.

## Data Availability

Data sharing is not applicable to this article as no data sets were generated or analyzed during the current study.
